# Molecular Pathways of Estrogen Receptor Action

**DOI:** 10.3390/ijms19092591

**Published:** 2018-08-31

**Authors:** Farzad Pakdel

**Affiliations:** Research Institute in Health, Environment and Occupation (Irset), Inserm U1085, Transcription, Environment and Cancer Group, University of Rennes 1, 35000 Rennes, France; farzad.pakdel@univ-rennes1.fr

The estrogen receptors (ERs) are typical members of the superfamily of nuclear receptors that includes the receptors that mediate the effects of steroid hormones, thyroid hormones, retinoid and vitamin D, as well as numerous orphan receptors. ERs, as other steroid receptors, mainly function as ligand-inducible transcription factors which bind chromatin, as homodimers, at specific response elements. It should also be noted that a tight reciprocal coupling between rapid ‘non-genomic’ and ‘genomic’ biological responses to estrogen occurs in many physiological processes. ERs have long been evaluated for their roles in controlling the expression of genes involved in vital cellular processes such as proliferation, apoptosis and differentiation. Given the various and pleiotropic functions of ERs, the dysregulation of their pathways contributes to several diseases such as, the hormone-dependent breast, endometrial and ovarian cancers as well as neurodegenerative diseases, cardiovascular diseases and osteoporosis. Several classes of ER ligands with agonist or antagonist activities in different E2-target tissues have been characterized. Moreover, ER ligands that efficiently block tumor growth and kill cancer cells have been developed.

In this special issue, “Molecular Pathways of Estrogen Receptor Action”, promising results in understanding the mechanisms underlying ER-mediated effects in various pathophysiological processes are represented, covering different roles of ER pathways in the tumorigenesis, the resistance to endocrine therapy, the dynamics of 3D genome organization and the cross-talk with other signaling pathways.

A key step in the physiological processes is the regulation of the transcriptional dynamics of gene networks. The article by Le Dily and Beato [1] summarizes the restructuration and chromatin folding during steroid hormone exposure, as well as the influence of three-dimensional genome organization in the response to steroid hormones. Deciphering these events may particularly be important to understand cell transformation and its progression in cancers where the genome is often rearranged during tumorigenesis. In addition, Yang et al. [2] update the effect of hypoxia on ER function in breast cancer. They focus on the link between ERs, the hypoxia inducible factor 1 and the histone lysine demethylase KDM4B, an important epigenetic modifier in cancer. Additionally, Saito and Cui [3] describe a possible cross-talk via transcriptional regulation between ERs and the estrogen-related receptors (ERRs) that partially share common target genes. Moreover, ERs can directly regulate the expression of genes encoding ERRs through the estrogen-response element within the promoter region. As ERRα is at the center of the coordination of transcriptional networks for neuronal and adaptive responses, this can potentially explain estrogenic actions in social behavior. Further, Hsu et al. [4] provide an overview of the possible role of ERs in lung cancer. Different aspects of the disease development, clinical studies, effects of tobacco smoking and environmental estrogens as well as ER activation and interactions with EGFR (epidermal growth factor receptor) are discussed. A critical review on the natural human anti-ERα antibodies capable of inducing estrogenic responses in breast cancer cells is given by Guy Leclercq [5]. These observations, not much mentioned previously, were recently confirmed and have been extended to autoimmune diseases. These data will open new paths to develop new strategies and to combine immunological and endocrine approaches for the management of breast cancer. The mechanism of action of these antibodies is also addressed.

In addition to cancerous cells, the non-cancer cells including tumor microenvironment (TME) are critical mediators of tumor progression. Besides the intracellular signaling, the interactions between cancer cells, stromal cells, immune cells, and extracellular molecules within the TME greatly impact antitumor immunity and the immunotherapeutic response. The potential role of estrogen signaling pathway, as a regulator of tumor immune responses, in the tumor microenvironment is discussed and reviewed by Rothenberger et al. [6]. Radiation therapy is widely used as one of the most common and effective therapeutic strategies. Nevertheless, the effect of ionizing radiation on the expression of ERs and ER signaling pathways in cancerous tissues, as well as on the endocrine therapy is not well-known. This topic is reviewed and discussed by Rong et al. [7]. They also summarize basic, pre-clinical and clinical studies that assess the consequences of anti-estrogen treatments in combination with radiotherapy in cancer.

There is an important link between estrogen signaling pathways and the regulation of the cardiovascular and immune systems. Trenti et al. [8] review the current understanding of the protective effects of estrogen on the cardiovascular system, including promoting endothelial healing and angiogenesis. They also describe the actions of estrogens in the immune function of the monocyte-macrophage system, through different pathways and in particular with regard to the production of cytokines. Recent studies have also suggested that estrogens exert their vascular protective effects, at least in part, through microRNA activity. Pérez-Cremades et al. [9] focus on the recent progress in determining the roles of estrogen-regulated microRNAs and their contribution in vascular biology. They summarize the microRNAs involved in estrogen action and the major role played by miR-23a and miR-22. However, further works focused on characterizing the role of estradiol-mediated miRNAs involved in vascular function are needed. Wnuk and Kajta [10] highlight the role of steroid and xenobiotic receptor signaling in apoptosis and autophagy of the central nervous system, and their potential implications in brain diseases. Finally, Lecomte et al. [11] discuss and summarize the in vitro and in vivo effects of phytochemicals interacting with ERs and their potential role in human health. The diversity of the mechanisms of action and the subtle balance between beneficial and harmful biological outcomes are also given.

In addition to the reviews mentioned above, eight research articles are included in this special issue. A clinical study reported by Matta et al. [12] describes a substantial variability in DNA repair capacity among breast cancer subtypes and suggests lowest repairs in triple negative breast cancer. Cardoso et al. [13] report estrogen metabolism-associated CYP2D6 and IL6-174G/C polymorphisms in Schistosoma haematobium Infection. From a primary culture approach, Kranc et al. [14] analyze the expression profile of genes regulating steroid biosynthesis and metabolism in human ovarian granulosa cells. An in vivo study conducted by d’Adesky et al. [15] indicates that nicotine modifies ER-β-regulated inflammasome activity and aggravates ischemic brain damage in female rats. The study conducted by Casanova-Nakayama et al. [16] examines the immune-specific expression and estrogenic regulation of the four ER isoforms in female rainbow trout. Alexandre-Pires et al. [17] evaluate functional aspects of sheep inguinal sinus gland and the mRNA and protein expressions of several hormone receptors including ERs. An in vivo and in vitro study conducted by Hinfray et al. [18] provides evidence regarding antagonistic effects of estradiol and genistein in combination using mixture concentration-response modeling in zebrafish. Serra et al. [19] report that triclosan lacks (anti-)estrogenic effects in zebrafish cells but alters estrogen response in zebrafish embryos.

While much remains to be learned, this special issue provides a background of the molecular mechanisms of ERs that is needed in clinical studies against estrogen-related diseases. Lastly, I would like to thank all the authors and referees for their efforts in supporting this special issue.
